# Comparison of Repeatability and Agreement between Swept-Source Optical Biometry and Dual-Scheimpflug Topography

**DOI:** 10.1155/2017/1516395

**Published:** 2017-12-10

**Authors:** Soyeon Jung, Hee Seung Chin, Na Rae Kim, Kang Won Lee, Ji Won Jung

**Affiliations:** Department of Ophthalmology and Inha Vision Science Laboratory, Inha University School of Medicine, Incheon, Republic of Korea

## Abstract

**Purpose:**

To assess the repeatability and agreement of parameters obtained with two biometers and to compare the predictability.

**Methods:**

Biometry was performed on 101 eyes with cataract using the IOLMaster 700 and the Galilei G6. Three measurements were obtained per eye with each device, and repeatability was evaluated. The axial length (AL), anterior chamber depth (ACD), keratometry (K), white-to-white (WTW) corneal diameter, central corneal thickness (CCT), and lens thickness (LT) were measured and postoperative predictability was compared.

**Results:**

Measurements could not be obtained with the IOLMaster 700 in one eye and in seven eyes with the Galilei G6 due to dense cataract. Both the IOLMaster 700 and Galilei G6 showed good repeatability, although the IOLMaster 700 showed better repeatability than the Galilei G6. There were no statistically significant differences in AL, ACD, steepest K, WTW, and LT (*P* > 0.050), although flattest K, mean K, and CCT differed (*P* < 0.050). The proportion of eyes with an absolute prediction error within 0.5 D was 85.0% for the IOLMaster 700 and was 80.0% for the Galilei G6 based on the SRK/T formula.

**Conclusions:**

Two biometers showed high repeatability and relatively good agreements. The swept-source optical biometer demonstrated better repeatability, penetration, and an overall lower prediction error.

## 1. Introduction

Accurate biometry measurements are the most crucial aspect of cataract surgery, because accurate measurement is the foundation of calculating intraocular lens (IOL) power. To improve the accuracy of biometry measurements, new devices have recently been introduced into clinical practice [1].

The most recently developed IOLMaster 700 (Carl Zeiss Meditec AG, Jena, Germany) has adopted swept-source optical coherence tomography (SS-OCT) technology, which uses a rapid-cycle and tunable wavelength laser light source to scan the eye, improving penetration and image quality [2–5]. The IOLMaster 700 enables not only biometric measurements, such as corneal keratometry (K), central corneal thickness (CCT), anterior chamber depth (ACD), white-to-white (WTW) corneal diameter, axial length (AL), and lens thickness (LT), but also detection of abnormal structure, such as lens dislocation or insufficient fixation [6–8].

Another new device, the Galilei G6 Lens professional (Ziemer Ophthalmic Systems AG, Port, Switzerland) is an optical biometry instrument that combines a dual rotating Scheimpflug camera, a placido disc topographer, and an optical coherence tomography-based A scan [9–11]. It performs axial biometry using light of 880 nm wavelength and which is based on low coherence interferometry [12]. The combination of the biometric measurement and anterior segment measurements with the Galilei G6 provides the intraocular lens (IOL) calculation [12].

As new devices have been introduced into clinical practice, it is important to evaluate the performance of these devices in clinical practice. Therefore, the aim of this study was [1] to compare the within-session repeatability of the two latest devices, IOLMaster 700 and Galilei G6 and [2] to evaluate the agreement of biometric parameters, including AL, ACD, K, WTW, CCT, and LT, and [3] to compare the IOL power calculation and predictability between the IOLMaster 700 and Galilei G6.

## 2. Materials and Methods

The eyes of patients, who had visited Inha University Hospital for cataract surgery, were included in this study. The study adhered to the tenets of the Declaration of Helsinki, and the prospective study protocol was approved by the institutional review board of Inha University Hospital. Informed consent was obtained from all the patients after the purpose and possible consequences of the study were explained to them. Subjects who were older than 20 years were included and none of these subjects had any histories of ocular disease, other than cataract, previous ocular surgery, or general disorders that affected the eye.

A single well-trained examiner examined all subjects using the IOLMaster 700 and Galilei G6. The order of the devices used for measurement was randomized. Before the examination, all patients were asked to completely blink before each scan was taken. Three measurements per eye were obtained by the same operator for each machine. Measurements included AL; ACD; K: flattest K (Kf), steepest K (Ks), and mean K (Km); WTW; CCT; and LT. The IOL power was calculated using the following three formulas: SRK/T, Hoffer Q, and Haigis. One surgeon performed surgery with implantation of the IOL in the capsular bag using one type of hydrophobic 1-piece monofocal aspheric IOL: the Tecnis ZCB00 (Abbott Medical Optics Inc., Santa Ana, CA, USA). The A-constants used for the IOL power calculations was based on the manufacturer's recommendations, with the target of emmetropia. The postoperative final refraction was performed with manifested refraction 4−6 weeks after cataract surgery.

### 2.1. Statistical Analysis

For repeatability analysis of two new devices, the within-subject standard deviation (Sw), coefficient of variation (CoV), and intraclass correlation coefficient (ICC) were evaluated from three repeated measurements from each instrument. The Sw was calculated using the square root of the residual mean square of repeated-measures analysis of variance, and CoV was calculated as the Sw divided by the average of the measurement and was expressed as a percentage; lower Sw and CoV represented higher repeatability. The ICC values were indicated as high (>0.90), moderate (0.75 to 0.90), and poor (<0.75).

The normality of the data was assessed with the Shapiro−Wilk test, and all ocular parameters were normally distributed. The mean of the biometric measurements was compared with a paired *t*-test. Agreements between the measurements using two devices were calculated using a Bland−Altman plot, and the 95% limit of agreement (LoA) of all parameters was recorded.

To compare the predictability between the two biometers, 40 eyes of 30 patients who underwent cataract surgery, were analyzed. The expected refractive results were compared using the obtained refractive error. The mean absolute prediction error was defined as the average absolute value of the numeric error (the final postoperative spherical equivalent (SE) minus the predicted postoperative SE). Statistical analyses were performed using the SPSS statistical software package (version 20.0; SPSS Inc., Chicago, IL) and *P* values less than 0.05 were considered to be statistically significant.

## 3. Results

A total of 101 eyes of 54 patients with a mean age of 60.4 years ± 9.6 (SD) were enrolled in this study. The measurements could not be obtained with the Galilei G6 in 7 (6.5%) eyes (dense cataract), whereas it could not be obtained with the IOLMaster 700 in 1 (0.9%) eye. These seven eyes were excluded from the analysis.

Table 1 shows the repeatability of measurements of various biometric parameters with two devices, the IOLMaster 700 and Galilei G6. Although, the repeatability of each device did not show statistically significant differences (all *P* > 0.050), the within-subject SD, CoV, and ICC for the AL, ACD, keratometric values, CCT, and LT measurements were superior with the IOLMaster 700 than with the Galilei G6.

**Figure 1:**
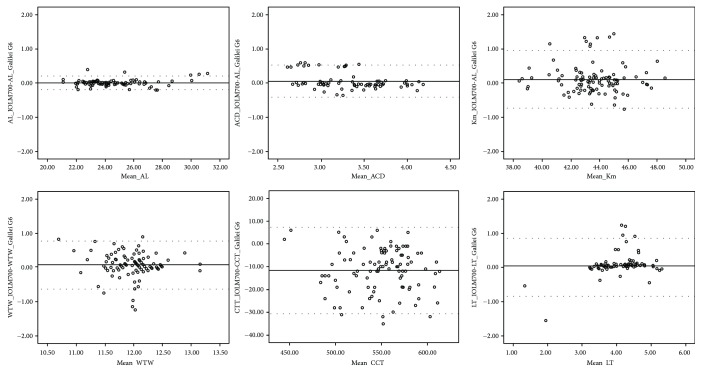
Bland−Altman plots showing the agreements between two biometers for axial length (AL), anterior chamber depth (ACD), mean keratometry (Km), white-to-white (WTW) corneal diameter, central corneal thickness (CCT), and lens thickness (LT) in the study subjects. The solid line represents the mean difference, and the dotted lines represent the 95% limits of agreement (LoA).

Table 2 shows the comparison of biometric measurements with the IOLMaster 700 and Galilei G6. Two biometers provided comparable mean AL measurements (*P* = 0.413), and the differences of ACD, Ks, WTW, and LT measurements were not statistically significant (*P* > 0.050). The keratometric values (Kf and Km) and CCT measurements were significantly different between the two devices (*P* < 0.050).  Figure 1 demonstrates the Bland−Altman plots for the agreement of various parameters between the two biometers. The agreement of AL measurements was better than those of other parameters (95% LoA, −0.19 to 0.21 in AL).

The mean absolute prediction errors obtained with the IOLMaster 700 and Galilei G6 were 0.31 ± 0.29 D and 0.31 ± 0.28 D for the SRK/T formula (*P* = 0.825), 0.29 ± 0.19 D and 0.28 ± 0.26 D for the Hoffer Q formula (*P* = 0.679), and 0.27 ± 0.18 D and 0.38 ± 0.27 D for the Hagis formula (*P* = 0.090). For the IOLMaster 700, the proportion of eyes within 0.5 D was 85.0% (34 eyes), and it was 80.0% (32 eyes) for the Galilei G6 based on the SRK/T formula. The proportion of eyes within 1.00 D of target was 100.0% for each of these devices (Table 3).

## 4. Discussion

In terms of the within-session repeatability of these devices, both the IOLMaster 700 and Galilei G6 showed good repeatability, and the CoV of all biometric parameters as determined with both biometers was less than 1%. The within-session repeatability of all parameters, except WTW measured by the IOLMaster 700, was better than that of Galilei G6. In particular, the AL is crucial for postoperative refractive errors after cataract surgery [13]; therefore, precise and repeatable AL measurement is important. In our study, the AL measured by the IOLMaster 700 showed excellent repeatability (CoV 0.021%, ICC 1.000), as compared to that of the Galilei G6 (CoV 0.147%, ICC 0.999); this result was very similar to those of previous studies [3, 6–9]. In a previous study comparing the IOLMaster 700 and IOLMaster 500, the repeatability and reproducibility of the swept-source device (IOLMaster 700) were evaluated in comparison with a TD-OCT (IOLMaster 500), and the ICC values were found to be high for all ocular biometry parameters (ICC 0.93−1.00) [3]. In this study, all parameters measured using IOLMaster 700, except keratometry and WTW, were 0.99 to 1.00 in ICC. Our present study also showed high ICC values (>0.99) for all parameters, except for Kf and WTW. Keratometry and WTW, which were determined using an LED light source, showed slightly lower ICCs than those of AL, ACD, CCT, and LT in another study [3]. Grulkowski et al. [6] also reported that SS-OCT had high repeatability for all biometric parameters (ICC > 0.99). Recently, the repeatability of SS-OCT was compared with that of an optical low-coherence reflectometry biometer (Lenstar 900) and the SS-OCT instrument demonstrated lower variability than that of the Lenstar 900 (CoV for AL, 0.05% in SS-OCT and 0.21% in Lenstar 900) [7]. Because SS-OCT device uses a rapid-cycle tunable laser source to scan the eye and there is no movement of the mirror [3], high repeatability of this instrument could be explained by this. Additionally, the repeatability of Galiliei G6 was proven in a previous study [9], which demonstrated that the AL measured by the Galilei G6 showed excellent repeatability (CoV 0.3%, ICC 0.996). Our data also had high ICC values (>0.96) for all parameters measured by the Galiliei G6, except WTW.

When comparing the biometric parameters between the IOLMaster 700 and the Galilei G6, there were no statistically significant differences, except for flattest K, mean K, and CCT. To date, there has been no study comparing the agreement between the IOLMaster 700 and Galilei G6. However, a few studies on the agreement between the IOLMaster 700 or Galilei G6 and other devices have recently reported that these two devices show excellent agreement with other devices [3, 7–9]. Agreement between a newer type of SS-OCT, the IOLMaster 700, and a standard partial coherence interferometry biometer, the IOLMaster 500, was demonstrated by previous studies [3, 8]. The SS-OCT biometer also measured the AL with fewer measurement failures than achieved with an optical low-coherence reflectometry (OLCR), the Lenstar 900; 96% measurements were successful with the SS-OCT, whereas 72% were successful with the OLCR [7]. In this study, the mean difference in the AL measured by the SS-OCT and the OLCR biometer was 0.01 mm, which would lead to a difference of about 0.02 D refractive error, which would not be clinically significant. Because the Galilei G6 is based on low-coherence reflectometry, Shin et al. [9] also recently demonstrated that the agreements in the AL between the Galilei G6 and Lenstar 900 were clinically good (95% LoA, −0.15 to 0.25 mm). In accordance with these results, the mean difference in the AL measurement was 0.01 mm and the agreement between the SS-OCT and the OLCR biometer was relatively good (95% LoA, −0.19 to 0.21 mm) in our present study.

Although the differences in the flattest K and mean K were statistically significant in our study, the actual differences were small, and possibly clinically insignificant. In a previous study [14], the mean K obtained with the Galilei G6 and the IOLMaster 500 showed statistically significant differences. The IOLMaster 700 uses a distance-independent telecentric keratometer for the keratometry measurement, as does the IOLMaster 500, and keratometry values are measured in 32 points arranged in 2 concentric rings of 1.65 mm and 2.30 mm in diameter [7]. While the Galilei G6 uses SimK data measured by placido-based corneal topography. The significant difference between these two devices could be explained by the different methods of keratometry measurement. The CCT in the present study also revealed significant differences between the two devices, and the range of 95% LoA was clinically wide. The results of CCT are not interchangeable, and the difference could be explained by the differences in detection methodology and light source variations [3, 15].

The prediction error in the 40 eyes that underwent cataract surgery was lower with the IOLMaster 700 than with the Galilei G6, but the difference was not statically significant. However, the proportion of eyes with an absolute prediction error within 0.5 D was 85.0% for the IOLMaster 700 and was 80.0% for the Galilei G6 based on the SRK/T formula. Comparison with previous reports is not possible, because no previous study has compared the IOLMaster 700 and the Galilei G6. Because our study sample size for postoperative results was small, further studies are needed.

In this study, 6 eyes with dense cataract could only be measured using the IOLMaster 700, and one eye with dense cataracts with posterior subcapsular cataract could not be measured by either of the two devices. This finding revealed that the SS-OCT optical biometer penetrated the opaque media better than did low coherence interferometry. The higher penetrability of IOLM 700 might be caused by the difference in the light source and scanning pattern [3]. The IOLMaster 700 uses a 1055 nm tunable laser source, whereas the Galilei G6 uses a 880 nm wavelength [16, 17]. The longer wavelengths penetrate tissue better and with less scatter [18, 19]. The SS-OCT optical biometer also uses an arc scan pattern for biometric measurements [20]; this might improve the penetration ability of the SS-OCT device [3].

In conclusion, the repeatability of all parameters, except WTW measured by the IOLMaster 700, was better than those measured by the Galilei G6, although both new devices provided highly repeatable measurements. The agreements of ocular biometric parameters, except keratometry and CCT between the IOLMaster 700 and Galilei G6, were good. The prediction error with IOLMaster 700 was lower and showed better penetration in this study.

## Figures and Tables

**Table 1:** Repeatability of various biometric parameters with two biometry devices.

Parameters	Within-subject standard deviation		Coefficient of variation (%)		ICC (95% CI)	
	IOLMaster 700	Galilei G6	IOLMaster 700	Galilei G6	IOLMaster 700	Galilei G6
AL (mm)	0.005	0.037	0.021	0.147	1.000 (1.000-1.000)	0.999 (0.998–0.999)
ACD (mm)	0.008	0.018	0.231	0.568	0.999 (0.998–0.999)	0.996 (0.993–0.997)
Kf (D)	0.098	0.282	0.228	0.664	0.989 (0.984–0.992)	0.956 (0.938–0.970)
Ks (D)	0.076	0.202	0.172	0.458	0.997 (0.996–0.998)	0.980 (0.971–0.986)
Km (D)	0.069	0.210	0.158	0.486	0.996 (0.994–0.997)	0.973 (0.962–0.982)
WTW (mm)	0.110	0.091	0.854	0.768	0.879 (0.838–0.912)	0.832 (0.769–0.882)
CCT (*μ*m)	1.873	4.148	0.349	0.731	0.995 (0.993–0.996)	0.965 (0.950–0.976)
LT (mm)	0.011	0.04	0.332	0.983	0.999 (0.999–0.999)	0.984 (0.977–0.990)

AL: axial length; ACD: anterior chamber depth; Kf: flat keratometry; Ks: steep keratometry; Km: mean keratometry; WTW: white-to-white corneal diameter; CCT: central corneal thickness; LT: lens thickness; ICC: intraclass correlation coefficient.

**Table 2:** Comparison of biometric measurements obtained using two new optical biometry devices in the study subjects.

Parameters	IOLMaster 700	Galilei G6	*P* value^∗^	Mean difference ± SD	95% limits of agreement^†^
AL (mm)	24.54 ± 2.08	24.53 ± 2.07	0.413	0.01 ± 0.10	−0.19, 0.21
ACD (mm)	3.35 ± 0.39	3.31 ± 0.47	0.115	0.05 ± 0.24	−0.42, 0.52
Kf (D)	43.10 ± 2.07	42.95 ± 2.04	**0.012**	0.15 ± 0.58	−0.99, 1.29
Ks (D)	44.21 ± 2.08	44.13 ± 2.08	0.116	0.08 ± 0.51	−0.91, 1.08
Km (D)	43.65 ± 2.01	43.54 ± 2.03	**0.008**	0.11 ± 0.43	−0.73, 0.95
WTW (mm)	11.98 ± 0.41	11.92 ± 0.45	0.058	0.07 ± 0.36	−0.64, 0.76
CCT (*μ*m)	541.27 ± 33.95	552.95 ± 34.35	**<0.001**	−11.68 ± 9.72	−30.73, 7.37
LT (mm)	4.12 ± 0.79	4.07 ± 0.62	0.352	0.04 ± 0.43	−0.80, 0.88

^∗^Using a paired *t*-test in the case of normally distributed variables and by the Wilcoxon signed-rank test in the case of nonnormally distributed variables. ^†^Limits of agreement is defined as mean difference ± 1.96 standard deviations. SD: standard deviation; AL: axial length; ACD: anterior chamber depth; Kf: flat keratometry; Ks: steep keratometry; Km: mean keratometry; WTW: white-to-white corneal diameter; CCT: central corneal thickness; LT: lens thickness.

**Table 3:** Comparison of prediction error among two biometry devices in subjects who underwent cataract surgery (*n* = 40 eyes).

Biometer	Formula	Prediction error (diopter)		Eyes within (%)		
		PE	Absolute PE	0.5 D	1.0 D	1.5 D
IOLMaster 700	SRK/T	−0.18 ± 0.38	0.31 ± 0.29	85.0	100.0	100.0
	Hoffer Q	−0.08 ± 0.34	0.29 ± 0.19	85.0	100.0	100.0
	Hagis	−0.15 ± 0.29	0.27 ± 0.18	90.0	100.0	100.0
Galilei G6	SRK/T	−0.23 ± 0.35	0.31 ± 0.28	80.0	100.0	100.0
	Hoffer Q	−0.16 ± 0.35	0.28 ± 0.26	77.5	100.0	100.0
	Hagis	−0.19 ± 0.43	0.38 ± 0.27	72.5	100.0	100.0

PE: prediction error.
